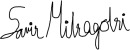# Letter from the Editor

**DOI:** 10.1002/btm2.10009

**Published:** 2016-06-03

**Authors:** Samir Mitragotri

**Affiliations:** ^1^ Dept. of Chemical Engineering Center for Bioengineering University of California Santa Barbara CA 93106

Dear Colleagues,

I am pleased to announce the launch of *Bioengineering & Translational Medicine*, a new journal from AIChE and SBE, and our publishing partner, Wiley. This new journal will focus on ways Chemical and Biological Engineering drive innovations and solutions that impact clinical practice and commercial healthcare products. The journal will also highlight scientific and technical breakthroughs currently in the process of clinical and commercial translation.

Like me, in recent years, you have likely seen reports in the literature about a large number of engineering advances that claim potential application in clinical medicine. Converting these discoveries into useful clinical products, however, is a major challenge. It requires attention to issues such as safety, manufacturability, regulatory hurdles, cost, and patient acceptance—issues that are not typically considered in academic research. Clinical translation also often requires simultaneous consideration of commercial translation, with the team and the resources required for clinical translation often also needed for any commercial translation. This interplay brings its own set of challenges. *Bioengineering & Translational Medicine* welcomes manuscripts that provide new insights into translational hurdles, as well as examples of technologies that have demonstrated excellent progress toward clinical or commercial translation. We also welcome manuscripts that provide new scientific insights and fundamental advances that facilitate translation.

*Bioengineering & Translational Medicine* also invites contributions on topics including, but not limited to, drug delivery, drug discovery, tissue engineering, synthetic biology, gene therapy, computational modeling and bioinformatics, among others. A more comprehensive list of topics can be found in the journal's statement of scope.

To researchers who are active, or aspiring to be active, in clinical and commercial translation of biological technologies, I say “this is your journal.” With a stellar advisory board behind us, we are poised for an exciting, quick, and successful launch. We look forward to receiving your manuscripts. We promise a rapid and fair review and fast publication. We also welcome your suggestions and comments on ways we can make the journal better serve your needs.

Thank you for sharing our enthusiasm about *Bioengineering & Translational Medicine* and I look forward to working with you.

Sincerely,